# High-Performance Fluorine-Lean Thin Aromatic Hydrocarbon Membranes Based on Polyvinylidene Fluoride for Hydrogen Fuel Cells

**DOI:** 10.3390/membranes14120263

**Published:** 2024-12-07

**Authors:** Tamas Nemeth, Zongyi Han, Lorenz Gubler

**Affiliations:** 1 PSI Center for Energy and Environmental Sciences, 5232 Villigen PSI, Switzerland; tamas.nemeth@psi.ch (T.N.); zongyi.han@psi.ch (Z.H.); 2 Sustainable Energy Technologies, SINTEF Industry, 7034 Trondheim, Norway

**Keywords:** fuel cells, proton exchange membranes, aromatic hydrocarbons, antioxidants

## Abstract

The impeding ban on per- and polyfluoroalkyl substances (PFAS) prompted researchers to focus on hydrocarbon-based materials as constituents of next-generation proton exchange membranes (PEMs) for polymer electrolyte fuel cells (PEFCs). Here, we report on the fuel cell performance and durability of fluorine-lean PEMs prepared by the post-sulfonation of co-grafted α-methylstyrene (AMS) and 2-methylene glutaronitrile (MGN) monomers into preirradiated 12 µm polyvinylidene fluoride (PVDF) base film. The membranes were subjected to two distinctly different accelerated stress test (AST) protocols performed at open-circuit voltage (OCV): the US Department of Energy-similar chemical AST (90 °C, 30% relative humidity (RH), H_2_/air, 1 bar_a_), developed originally for perfluoroalkylsulfonic acid (PFSA) membranes, and the high relative humidity AST (80 °C, 100% RH, H_2_/O_2_, 2.5 bar_a_), designed for aromatic hydrocarbon membranes. We found that doping the grafted membranes with a metalated porphyrin antioxidant can simultaneously reduce membrane aging and improve fuel cell performance.

## 1. Introduction

Fuel cells (FCs) are electrochemical devices that directly convert chemical energy into electricity with high efficiency and zero emissions at the point of use. The widespread use of hydrogen FCs in vehicular transportation, especially in heavy duty trucks, aviation, and maritime sectors, can contribute to the decrease in greenhouse gas emissions, targeted by the “Net Zero by 2050” initiative [1]. The most common type of FC contains a proton-conducting proton exchange membrane (PEM) coated with catalyst layers (CLs), sandwiched between gas diffusion layers. The PEM acts as both electrolyte and separator for gases and electrons and enables operation at high current densities. State-of-the-art PEMs comprise perfluoroalkylsulfonic acid (PFSA) ionomers such as Nafion™ by Chemours (New Castle, DE, USA). They possess high chemical stability, yet high productions cost [2], poor gas barrier properties [3], and limited dimensional stability, currently impeding their widespread application [4]. These materials are “forever chemicals” that pose an environmental concern that prompted the European Union to consider banning their non-essential use [5].

Therefore, it is of general interest to develop partially or nonfluorinated membrane alternatives to address these limitations. Irradiation grafting is a versatile technique to obtain PEMs from various monomers [6]. First, a base polymer is exposed to high-energy ionizing radiation in the presence of air, leading to the formation of peroxide and hydroperoxide sites on the polymer. Upon heating, these peroxide groups decompose, generating free radicals, which can react in the next step with the chosen monomer(s) to obtain a grafted film [7,8,9]. Post-polymerization reactions can be performed to introduce the desired properties. In the case of FC applications, radiation-grafted membranes contain a mechanically and chemically robust base film, typically the fluoropolymer poly(ethylene-*co*-tetrafluoroethylene) (ETFE), and aromatic hydrocarbon-type grafted constituents [10]. Pre-irradiation grafting can also be used to prepare anion exchange membranes, which was pioneered by Slade and Varcoe [11,12]. The choice of the base film determines final membrane thickness and, with the grafted monomers, influences the final physico-chemical properties of the material. Rigorous optimization of graft constituents enabled membranes that could match the durability of certain Nafion-type PEMs [10], but performance still needs to be further improved. The conductivity and fuel cell performance of ETFE-based irradiation-grafted membranes are limited by the thickness of the base film; thicker membranes exhibit decreased conductivity [9]. Additionally, proton-conductive groups are typically introduced via the grafted constituents. Therefore, increasing the graft level improves fuel cell performance, although it may reduce mechanical stability [13].

Recent technoeconomic assessments highlight that widespread application of fuel cells is hindered by high manufacturing costs [14]. Presently, the cost contribution of PEM and CL components amounts to nearly half of the total production costs. One way to increase market penetration of the FC technology is to reduce costs by using ultra-thin PEMs. Here, the use of an ETFE base polymer poses a limitation, as at reduced thickness enhanced gas permeability is observed. The use of alternative base films can alleviate these issues. Polyvinylidene fluoride (PVDF) is a semi-crystalline fluoropolymer combining excellent chemical and mechanical properties, offering better processability and gas barrier properties than ETFE or perfluoropolymers [15,16].

In this study, we investigate thin (18–24 µm) PEMs, prepared by sulfonating films obtained by irradiation-grafting the monomers α-methylstyrene and 2-methylene glutaronitrile into a PVDF base film. The use of PVDF as base film for the preparation of radiation-grafted membranes has been previously reported by Chen et al. [17,18]. We compare the performance in the single fuel cell, and we report on the in situ fuel cell durability under various accelerated chemical-stress conditions complemented with extensive post-test analyses to quantify the extent of the degradation. In addition, we implement a strategy to combat oxidative aging, by doping the membranes with a metalated porphyrin-type antioxidant.

## 2. Results and Discussion

### 2.1. PEM Fabrication and Characterization

**Figure 1:**
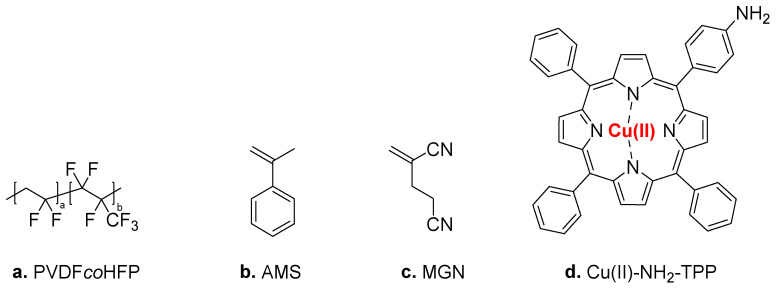
Chemical structure of (**a**) the PVDF base film, (**b**) the AMS, (**c**) MGN monomers, and of (**d**) Cu(II)-NH_2_-tetraphenylporphyrin (TPP), the radical repair agent used in this study.

**Scheme 1:**
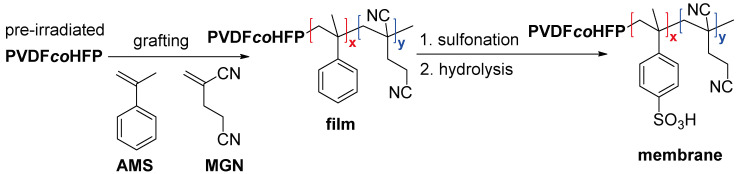
Preparation of the PVDF-g-poly(AMS-co-MGN) films.

To prepare novel poly(vinylidene fluoride-*co*-hexafluoropropylene)-based (PVDF) aromatic hydrocarbon containing PEMs (Figure 1), we followed the procedure described in Scheme 1. Commercial PVDF films with 12 µm thickness were selected as the base polymer for *e*-beam irradiation as PVDF has high resistance against radiation-induced mechanical degradation [7]. Moreover, it can be dissolved in aprotic polar organic solvents, such as dimethylformamide (DMF) and dimethylacetamide (DMAc), enabling solution phase post-polymerization modifications. Lastly, very thin PVDF films can be obtained without adversely affecting mechanical properties. During FC operation, thin PEMs enable effective back-diffusion of water from cathode to anode side and typically have lower membrane resistance, resulting in improved cell performance. The base films were pre-irradiated at room temperature under air atmosphere with a total dose of 25, 50, and 100 kGy, respectively. Next, the hydrocarbon monomers α-methylstyrene (AMS) and 2-methylene glutaronitrile (MGN) were grafted onto the base polymer to obtain PVDF-*g*-poly(AMS-*co*-MGN) films. Unlike styrene [19], the monomer AMS does not readily polymerize by the radical mechanism by itself [20]; therefore, we used MGN as co-monomer. AMS features an aromatic moiety that can be substituted with proton-conducting groups, whereas MGN was used as a comonomer to improve the low self-chain-growth of AMS. Additionally, MGN is known to improve the gas barrier properties of ion exchange membranes [21].

**Figure 2:**
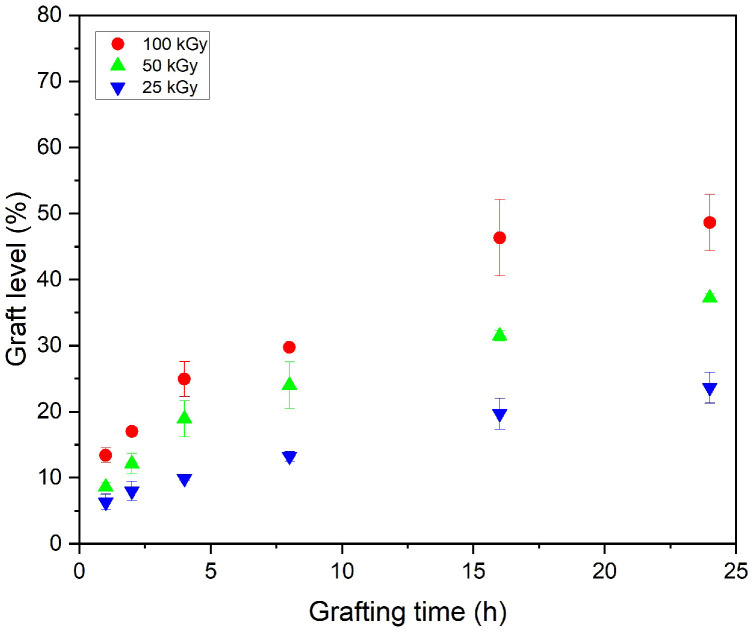
Reaction kinetics for co-grafting the monomers α-methylstyrene and 2-methylene glutaronitrile into 12 µm PVDF base film with irradiation doses of 25 (blue triangles), 50 (green triangles), and 100 kGy (red circles), at 65 °C.

The pre-irradiated PVDF films were immersed into the optimized grafting solution that contained 20 v% monomer mixture of AMS:MGN (1:1 molar ratio) in IPA and water solvent mixture (1:1 volumetric ratio). This solution was deaerated by bubbling N_2_ for at least 45 min, before placing the grafting reactors onto a preheated (65 °C) heating plate for a controlled time. The grafting kinetics, represented in Figure 2, indicate that, for all irradiation doses, the graft level (GL) reached a plateau after 15–25 h of reaction due to the reduced grafting reaction rate caused by the recombination of polymer chains and the decay of radicals. High graft levels could be obtained in IPA–water mixtures. IPA is a nonsolvent for the growing AMS-MGN-containing polymer chains, limiting their mobility and effectively prolonging the lifetime of chain-end radicals [22].

The obtained films were subjected to an electrophilic aromatic substitution (S_e_Ar) sulfonation reaction and subsequent hydrolysis, Scheme 1 and Reactions (1) and (2), to introduce the protogenic sulfonic acid groups [9]. Near theoretical yields were obtained upon using 5–10 v % chlorosulfonic acid in DCM.

Ar-H + ClSO_3_H → Ar-SO_2_Cl + H_2_O (1)

Ar-SO_2_Cl + H_2_O → Ar-SO_3_H + HCl (2)

**Figure 3:**
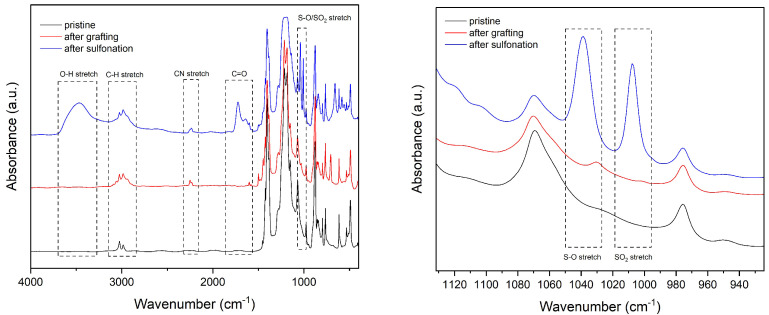
**Left**: transmission FT-IR spectra of pristine PVDF films (black), grafted films (red), and sulfonated membranes (blue). **Right**: expanded spectra of characteristic peaks for S-O and SO_2_ stretching vibration.

FT-IR analyses were performed to evaluate the grafting and sulfonation reactions (Figure 3) [23]. Successful grafting of AMS is indicated by the appearance of the aromatic and alkyl C-H stretching bands at 2900–3100 cm^−1^ and the aromatic C-C stretching bands between 1500 and 1600 cm^−1^ (Figure 3 left). In addition, the appearance of the nitrile stretch at 2230 cm^−1^ indicates the successful incorporation of the MGN monomer. The appearance of the S-O stretching vibration at 1039 cm^−1^ and of the SO_2_ symmetric stretch at 1009 cm^−1^ confirms successful sulfonation (Figure 3 right) of the AMS unit, yielding α-methylstyrene sulfonic acid (AMSSA) groups [20,24]. The presence of the C=O stretching peak at 1700 cm^−1^ indicates partial hydrolysis of the nitrile groups in MGN to amide or carboxylic acid groups.

**Figure 4:**
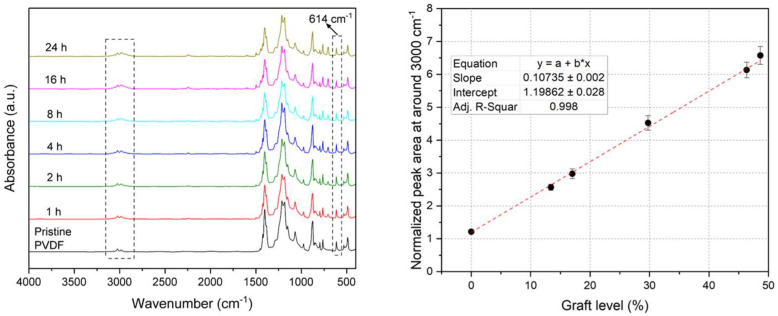
FTIR as a tool to quantify the graft level. **Left**: the peak area at around 3000 cm^−1^ is normalized to the peak area at around 614 cm^−1^. **Right**: the obtained linear correlation between the normalized peak area at around 3000 cm^−1^ and the graft level.

Furthermore, FT-IR can be used as a tool to quantify the graft level of unknown PVDF-based samples. Based on the relationships between the C–H stretch peak area at around 3000 cm^−1^ with the grafting time (Figure 4 left) and the graft level with the grafting time (grafting kinetics from Figure 2), the relationship between the peak area at around 3000 cm^−1^ and the graft level can be obtained (Figure 4 right). By normalizing the peak area at around 3000 cm^−1^ (due to C–H stretch vibrations) to the area of the peak at around 614 cm^−1^, which is a characteristic peak for PVDF films and only varies slightly during grafting [25], we can eventually obtain a linear relationship between the normalized peak area at around 3000 cm^−1^ and the graft level, as seen in Figure 4. For an AMS:MGN-grafted PVDF film with an unknown graft level, the FT-IR can be measured and the normalized peak area at around 3000 cm^−1^ (with respect to the peak area at around 614 cm^−1^) can be estimated. With this value and the linear fit shown in Figure 4 right, the graft level can then be evaluated.

**Figure 5:**
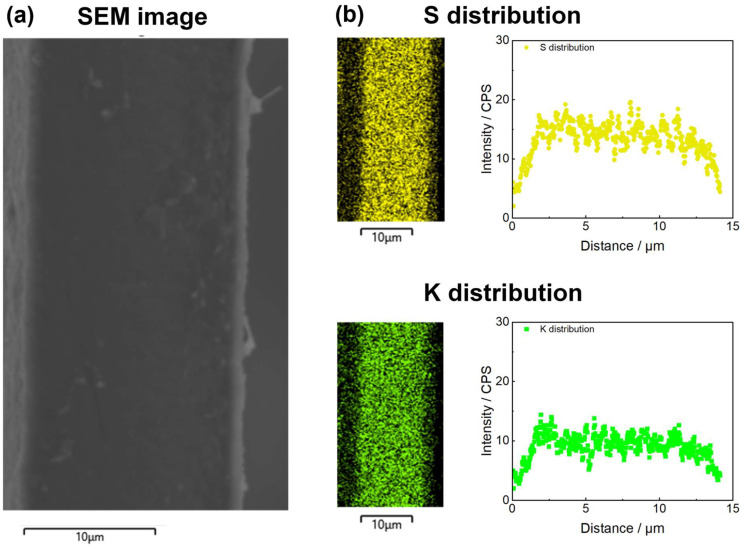
SEM image (**a**) and EDX mapping (**b**) of a cross-section of an ETFE-*g*-poly(AMSSA-*co*-MGN) membrane with a graft level of 37%.

Cross-section SEM-EDX mapping supplemented the FT-IR measurements. The even distribution of the elements sulfur and potassium across the thickness of the potassium-exchanged membranes suggests uniform grafting and sulfonation (Figure 5).

### 2.2. Fuel Cell Tests

For further ex situ characterization and FC tests, we selected membranes with a graft level of 43, 52, and 63%. Compared to PFSA-type PEMs, hydrocarbons require a higher ion exchange capacity to reach similar proton conductivity. This is due to their inherently lower acidity, insufficient microphase separation, and less dense polymeric backbone [26,27].

**Figure 6:**
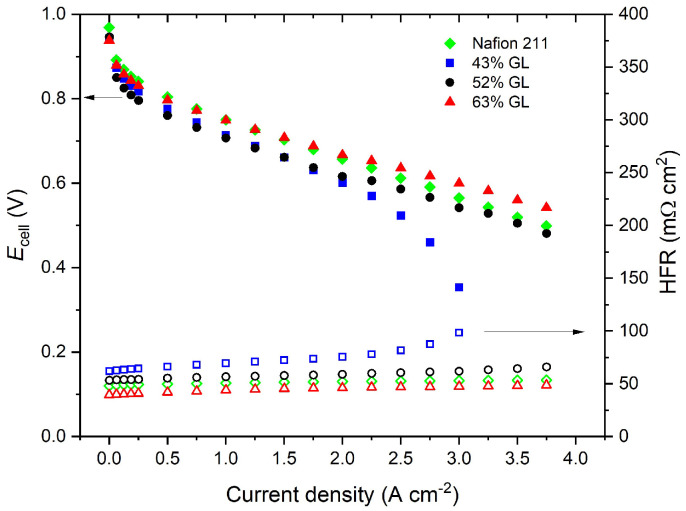
**Left** *y*-axis: polarization curves of the tested membranes, 43% GL (blue squares), 52% GL (black circles), 63% GL (red triangles), and of the reference N211 (green diamonds). **Right** *y*-axis: high-frequency resistance (HFR) of the tested membranes. Data were recorded at 80 °C with 2.5 bar_a_ back pressure, fully humidified gases at the inlet, a minimum flow of 200 mL_n_ min^−1^, and a stoichiometry of 1.5 for both H_2_ and O_2_. Commercial Pt-coated gas diffusion electrodes featuring a PFSA-based binder (Johnson Matthey ELE 0244, 0.4 mg Pt cm^−2^) were used.

We found that both in-plane conductivity and ion exchange capacity increase with GL ranging between 64–89 mS cm^−1^ and 1.23–1.54 mmol g^−1^, respectively (Table 1). Following the ex situ characterization, the PEMs were exchanged to proton form and sandwiched between commercial gas diffusion electrodes to obtain membrane electrode assemblies (MEAs, see details in Supplementary Materials) that were subjected to FC tests. Polarization curves were recorded galvanostatically at 80 °C cell temperature with 2.5 bar_a_ back pressure, fully humidified gases at the inlet, a minimum flow rate of 200 mL_n_ min^−1^ with a fixed stoichiometry of 1.5 for both H_2_ and O_2_ (Figure 6). Nafion™ 211 (N211) with similar dry thickness was used as reference. We found that the grafted PEMs became brittle during hot pressing; therefore, we adopted the wet-assembly method. Under these testing conditions, for all tested samples, the open-circuit voltage (OCV) was below the theoretical one, 1.196 V. This is due to H_2_ crossover to the cathode side, depolarizing the cathode potential, and a result of mixed potential of platinum oxidation and O_2_ reduction. With an increase in graft level, the performance of the grafted PEMs improved. At GL of 52%, the performance of N211 could be matched, whereas the 63% GL sample outperformed the reference (Figure 6). We hypothesize that, at a low GL of 43%, the through-plane conductivity of the samples is compromised, as a result of inadequate formation of proton-conductive channels. It is worth noting that, even with a low GL of 43%, our PVDF-based, AMS/MGN-grafted PEM still shows a significant performance improvement over a recently published sulfonated polystyrene-grafted PVDF-based membrane under similar testing conditions [28]. The novel PVDF-based membranes showed superior performance to fluorinated ethylene propylene (FEP)-based irradiation grafted membranes featuring styrene and divinylbenzene as grafted constituents [29].

After establishing the performance of the newly fabricated MEAs, we performed accelerated stress tests (ASTs) to evaluate in-device durability. First, we performed an AST developed for hydrocarbon PEMs by holding the cell at 80 °C cell temperature, under 2.5 bar_a_ back pressure and using fully humidified gases, H_2_ and O_2_, at the inlet until OCV decreased below 0.8 V [30].

**Figure 7:**
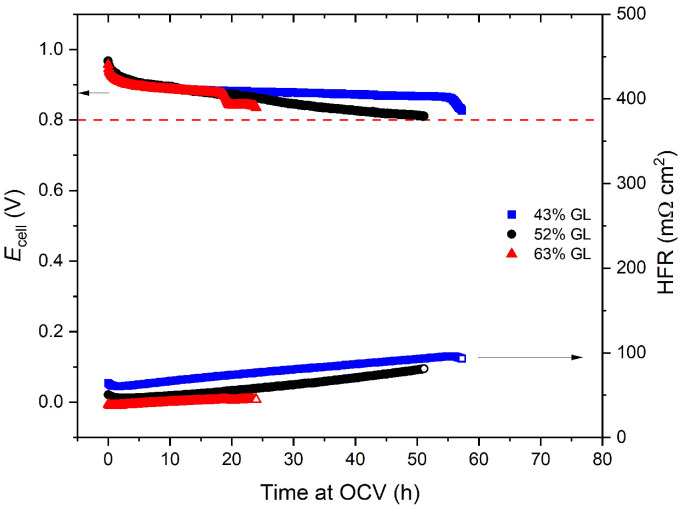
AST at high relative humidity: decay of cell potential (**left** *y*-axis) and changes in high-frequency resistance (**right** *y*-axis) of the tested membranes with 43% GL (blue squares), 52% GL (black circles), 63% GL (red triangles). Data were recorded at OCV, 80 °C with 2.5 bar_a_ back pressure and fully humidified H_2_ and O_2_ at the inlet.

In case of the AST performed at high relative humidity (Figure 7), all hydrocarbon PEMs failed within 60 h. Under these testing conditions, N211 can last for several hundred hours. We also observed a linear increase in the high-frequency resistance (HFR) during the AST that can be explained by the cleavage and wash-out of sulfonated aromatic constituents, leaving the more durable and non-conductive PVDF base film largely unaffected. We found that the lifetime decreased with increasing GL (Figure 7), also testified by differences in the rate of IEC loss (Table 1). The membrane with 43% GL had the best durability in the series, despite its lower thickness (Table 1). The BOT hydrogen crossover was measured and it was found that the membrane with the highest GL had the highest initial H_2_p; therefore, we hypothesize that, with an increase in GL, the water uptake also increases, which directly affects the gas barrier properties (Table 1). A higher crossover causes an increase in the rate of radical formation that occurs in the membrane electrode assembly in the presence of reactive gases (H_2_ and O_2_) and the noble metal catalyst. Formed radicals inflict chemical damage to the PEM, thereby limiting prolonged FC operation [31]. A GL of 52% offers a good compromise between performance and durability; therefore, in the following experiments, we studied this targeted GL in more detail.

**Figure 8:**
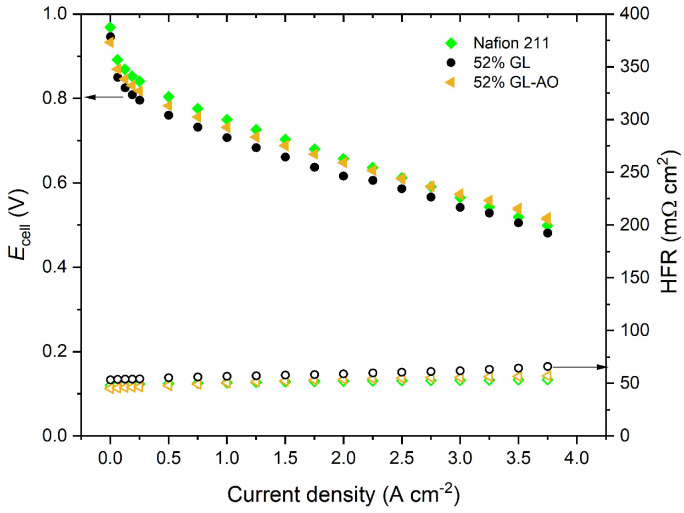
Effect of antioxidant doping on performance. Polarization curves of the tested membranes, 52% GL (black circles) *w*/*o* antioxidant and w/ antioxidant (golden triangles), and of the reference N211 (green diamonds). Data were recorded at 80 °C with 2.5 bar_a_ back pressure, fully humidified gases at the inlet, a minimum flow of 200 mL_n_ min^−1^, and a stoichiometry of 1.5 for both H_2_ and O_2_.

To improve in-device lifetime, we implemented a recently reported antioxidant strategy [30]. Briefly, during radical attack on aromatic hydrocarbon-type PEM constituents, oxidized species are formed [32]. These intermediates have a relatively long lifetime that enables reducing agents to “repair” these constituents. Here, we used a Cu(II)-porphyrin-type antioxidant (Figure 1) to dope the grafted PEMs (Scheme S1). We found that the antioxidant doping decreased both the BOT in-plane conductivity and IEC. This can be explained by the acid–base interaction between the sulfonic acid groups of the PEM and the protonated amino group of the antioxidant, decreasing the amount of available protogenic groups for proton conduction. We also observed that this decrease in conductivity or IEC did not impair in-cell performance; in fact, the modified PEM (52% GL-AO) outperformed the non-modified one (Figure 8).

**Figure 9:**
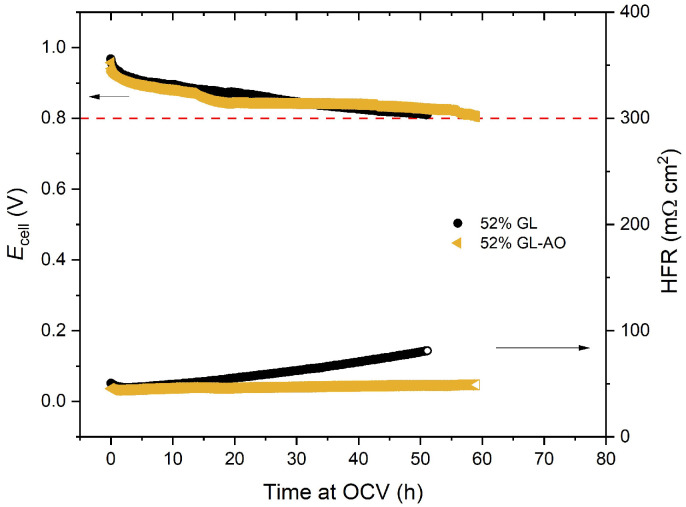
AST at high relative humidity: decay of cell potential (**left** *y*-axis) and changes in high-frequency resistance (**right** *y*-axis) of the tested membranes with 52% GL *w*/*o* doped antioxidant (black circles) and w/ doped antioxidant (golden triangles). Data were recorded at OCV, 80 °C with 2.5 bar_a_ back pressure and fully humidified H_2_ and O_2_ at the inlet.

Antioxidant doping enhanced the durability of the PEMs: lifetime increased by 20%, from 50 h to 60 h, in the AST performed at high relative humidity. In case of the non-modified membrane, HFR nearly doubled (BOT: 44 mOhm cm^2^, EOT: 81 mOhm cm^2^), whereas for the modified PEM it only increased by 10%, from 43 to 48 mOhm cm^2^ during the AST (Figure 9). Also, the IEC loss rate decreased by nearly 60% from 1.46 to 0.62% h^−1^ (Table 1). Durability of the novel PVDF-based PEMs is lower than ETFE-based membranes featuring covalently bound antioxidants [30,33].

**Figure 10:**
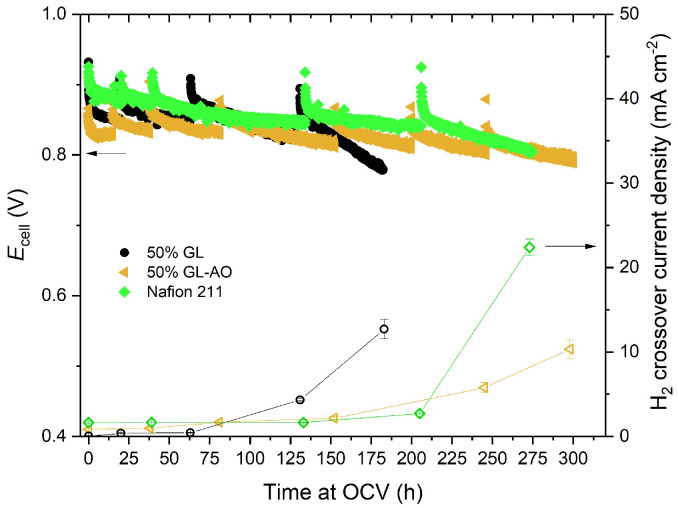
AST under DOE-like conditions. **Left** *y*-axis: decay of cell potential of the tested membranes with 50% GL *w*/*o* doped antioxidant (black circles), w/ doped antioxidant (golden triangles), and N211 reference. **Right** *y*-axis: changes in hydrogen crossover current density. Data were recorded at OCV, 90 °C at ambient pressure, 30% RH at the inlet for H_2_ and air.

Next, we applied a different AST protocol, similar to the one proposed by the US Department of Energy (DOE) that was originally developed for PFSA-type PEMs. We kept the cell at OCV, 90 °C and applied an RH of 30% on both the anode (H_2_) and cathode (air) side at ambient pressure until the failure criterion of a hydrogen crossover current density of >10 mA cm^−2^ was reached [34,35,36]. We prepared new membranes with a GL of 50%. Here, doping with antioxidant had no negative impact on the conductivity, whereas IEC decreased (Table 2). We included N211 as a reference in these tests. The non-modified PEM failed after 175 h, N211 after 275 h, and the modified membrane lasted for 300 h (Figure 10). The presence of the antioxidant caused a >35% decrease in the rate of IEC loss (Table 2). The reduced durability of N211 under these conditions is due to chain scission, an additional degradation mechanism for PFSA-type PEMs at low RH [37,38].

IEC, by definition, describes the amount of protons (or ions) exchanged per gram of dry polymer, typically expressed in milliequivalents per gram (meq g^−1^) or millimols per gram (mmol g^−1^). We hypothesize that the smaller IEC decrease rate for N211 may be explained by differences in the degradation mechanism compared to hydrocarbon PEMs [33,39,40]. For N211, the loss of the protogenic sulfonic acid groups is accompanied by pronounced membrane thinning [39], also indicated by the sharp increase in hydrogen permeability at the end of testing. Whereas for hydrocarbon membranes, the more pronounced IEC loss rate can be explained by sulfonic acid loss without significant material thinning [33,40], supported by the relatively smaller increase in permeability (Figure 10, Table 2).

Unlike in recent studies on covalently attached antioxidants, here the wash-out of the antioxidant may limit its effectiveness [30,33]. Therefore, by measuring the Cu-content in the PEMs before and after testing, we investigated the stability of the doped antioxidant in the strongly acidic and oxidizing environment of a fuel cell. We used X-ray fluorescence spectroscopy (XRF), a reported method to measure metal content in PEMs (Table 3, see further details in Supplementary Materials) [41].

We found that the Cu-content decreased in both ASTs, indicating wash-out of the antioxidant (Table 3). We estimated a 50% loss in the hydrocarbon-specific AST and a 70% decrease in the DOE-like AST. This can be explained by considering the differences in relative humidity (100% vs. 30%) and the testing durations (60 h vs. 300 h) of the hydrocarbon-specific and DOE-like ASTs.

## 3. Conclusions

Our study demonstrates that fluorine-lean PEMs, prepared by post-sulfonation of co-grafted α-methylstyrene (AMS) and 2-methylene glutaronitrile (MGN) monomers into a preirradiated 12 µm polyvinylidene fluoride (PVDF) base film, can match and exceed the fuel cell performance of N211.

We implemented an antioxidant strategy by doping the grafted PEMs with Cu-porphyrin and found that the modified membranes give excellent fuel cell performance, demonstrating that this stabilization approach does not adversely affect cell performance. We performed two types of ASTs: in a hydrocarbon-specific AST we operated the cell at high relative humidity and high partial pressure of oxygen until the OCV dropped below 0.8 V; in a DOE-like test, performed at low relative humidity and ambient pressure, we used the increase in hydrogen crossover beyond 10 mA cm^−2^ as a failure criterion. We found that, while the durability of the antioxidant-containing membranes improved, they still failed after 60 h of testing in the hydrocarbon-specific AST. We demonstrated that the modified membranes had a higher durability than N211 under DOE-like testing conditions. We succeeded in developing a method that is applicable for all PEMs, by simply doping the membranes with an antioxidant to mitigate radical-induced degradation.

## Figures and Tables

**Table 1:** Properties of the prepared PEMs with 43, 52, and 63% graft level, and of the reference Nafion™ 211.

Sample	Wet Thickness ^1^ (µm)	In-Plane Conductivity ^1^ (mS cm^−1^)	IEC_BOT_ (mmol g^−1^)	IEC_EOT_ (mmol g^−1^)	IEC Loss Rate ^2^ (% h^−1^)	H_2_p ^3^ (mA cm^−2^)	Water Uptake ^1^ (%)
N211	25.2 ± 0.5	55 ± 1	0.92 ± 0.02	0.88 ± 0.03	0.07 ± 0.06	2.9 ± 0.1	15 ± 2
43% GL	19.7 ± 0.2	64 ± 2	1.23 ± 0.04	0.37 ± 0.02	1.22 ± 0.03	1.4 ± 0.2	54 ± 7
52% GL	20.2 ± 1.7	77 ± 2	1.44 ± 0.02	0.15 ± 0.04	1.46 ± 0.04	1.8 ± 0.2	59 ± 1
63% GL	24.2 ± 0.2	89 ± 4	1.54 ± 0.07	0.73 ± 0.01	2.09 ± 0.09	4.5 ± 0.1	65 ± 10
52% GL-AO ^4^	20.3 ± 0.3	71 ± 2	1.33 ± 0.04	0.84 ± 0.01	0.62 ± 0.03	2.7 ± 0.2	75 ± 3

^1^ Measured at 25 °C. ^2^ Estimated from the beginning of test (BOT) and end of test (EOT) IEC, and the AST time. ^3^ Hydrogen crossover at BOT. ^4^ Doped with antioxidant.

**Table 2:** PEMs used in the DOE-like AST.

Sample	Wet Thickness ^1^ (µm)	In-Plane Conductivity ^1^ (mS cm^−1^)	IEC_BOT_ (mmol g^−1^)	IEC_EOT_ (mmol g^−1^)	IEC Loss Rate (% h^−1^)	H_2_p ^2^ (mA cm^−2^)
N211	25.2 ± 0.5	55 ± 1	0.92 ± 0.02	0.74 ± 0.04	0.07 ± 0.02	1.6 ± 0.1
50% GL	17.5 ± 0.5	58 ± 2	1.16 ± 0.05	0.46 ± 0.11	0.32 ± 0.05	<0.1 ± 0.1
50% GL-AO	19.0 ± <0.1	63 ± 6	1.07 ± 0.1	0.44 ± 0.08	0.20 ± 0.03	0.8 ± 0.1

^1^ Measured at 25 °C. ^2^ Hydrogen crossover at BOT.

**Table 3:** Changes in Cu-content determined by XRF method ^a^.

Type	Cu to S Ratio ^b^	Cu-Content/AU	AST Type
52% GL-AO at BOT	(5 ± 1) × 10^−3^	430 ± 60	Hydrocarbon-specific
52% GL-AO at EOT	(4 ± 1) × 10^−3^	210 ± 40	Hydrocarbon-specific
50% GL-AO at BOT	(5 ± <1) × 10^−3^	320 ± 50	DOE-like
50% GL-AO at EOT	(3 ± <1) × 10^−3^	100 ± 20	DOE-like

^a^ the estimation method is described in the Supplementary Materials. ^b^ calculated from the relative fluorescence intensity of each element (Cu K_α_ and K_β_ and S K_α_).

## Data Availability

Data is available from the corresponding author upon reasonable request.

## Supplementary Materials

The following supporting information can be downloaded at: https://www.mdpi.com/article/10.3390/membranes14120263/s1, Experimental details; FT-IR and XRF measurements of the tested membranes. Scheme S1: antioxidant doping reaction; Figure S1: color of the Cu(II)-porphyrin-containing PEM during the doping reaction.
