# Supplementary material for: High-Performance Fluorine-Lean Thin Aromatic Hydrocarbon Membranes Based on Polyvinylidene Fluoride for Hydrogen Fuel Cells
Source: Membranes (Basel). 2024 Dec 7;14(12):263. doi: 10.3390/membranes14120263 (PMC11679537; doi:10.3390/membranes14120263)
Supplement: Supplementary file 1 [file membranes-14-00263-s001.zip › membranes-3317831-supplementary.pdf]

High-Performance Fluorine-Lean Thin Aromatic Hydrocarbon Membranes Based on Polyvinylidene Fluoride for Hydrogen Fuel Cells

AUTHOR NAMES

Tamas Nemeth[1,2=], Zongyi Han[1=], Lorenz Gubler[1]*

AUTHOR ADDRESS

[1] PSI Center for Energy and Environmental Sciences, 5232 Villigen PSI, Switzerland

[2] Sustainable Energy Technologies, SINTEF Industry, 7034 Trondheim, Norway

lorenz.gubler@psi.ch

KEYWORDS fuel cells, proton exchange membranes, aromatic hydrocarbons, antioxidants

Table of Contents

Table of Contents 2
Experimental Procedures 3
Chemicals 3
Porphyrin synthesis 3
Synthesis of sulfonated AMS/MGN co-grafted membranes 3
Antioxidant doping 4
Ion exchange capacity (IEC) determination 5
Proton conductivity measurements 6
Membrane electrode assembly (MEA) preparation and fuel cell assembly 6
Fuel cell testing 7
Cell conditioning procedure 7
Polarization curve 7
Staircase voltammetry 8
Accelerated stress tests 8
Membrane post-test analysis 8
Fourier Transform Infrared Spectroscopy (FT-IR) 9
X-ray fluorescence spectroscopy (XRF) 9
Determination of the relative Cu(II) content by XRF 9

Experimental Procedures

Chemicals

α-methylstyrene (AMS, 99%, Sigma Aldrich), 2-methylene glutaronitrile (MGN, 95%, ABCR), chlorosulfonic acid (Sigma Aldrich) and sulfuric acid 95% (Fischer Scientific) was used as received and diluted as necessary. Sodium sulfate 99.0%, cerium(III) sulfate anhydrous 99.99% and hydrogen peroxide (30% (w/w) in H_2O) were obtained from Sigma Aldrich. Ultra-pure water was provided by a Milli-Q or Evoqua Ultra Clear UV Plus water purification system. PVDF*co*HFP base films with 12 μm thickness were provided by an industry partner. Nafion™ reference membranes were purchased from Chemours.

Porphyrin synthesis

The porphyrin derivative, copper(II)-5-(4-aminophenyl)-10,15,20-triphenylporphyrin was synthesized as described in the literature and then was used to dope the grafted membranes.[1]

Synthesis of sulfonated AMS/MGN co-grafted membranes

AMS was co-grafted together with MGN onto pre-irradiated PVDF base films according to the reaction scheme shown in **Scheme 1**. All grafting reactions were carried out in cylindrical glass reactors (3 cm diameter, 18 cm height, 60 mL capacity) which contained grafting solution and immersed base films under N_2 atmosphere. Optimized solution contained 20 v% monomer with AMS : MGN = 1:1 molar ratio in 1:1 (volumetric ratio) solution of IPA and water. Before starting the reaction, N_2 was bubbled through the grafting solution for at least 45 min to remove any dissolved O_2. The grafting reactions were then carried out by placing the reactors in a preheated dry block heater (65 °C) for a controlled grafting time. For the kinetics study of the grafting reactions, the grafting times were set to be 1, 2, 4, 8, 16, and 24 h and the grafting conditions were set to the earlier found optimum. For each reaction, after the set grafting time was reached, the reaction was quenched by rinsing the grafted film 2 times with isopropanol (IPA). And then the grafted film was put into dichloromethane (DCM) for 2 h to remove any homopolymer and

monomer residues. Finally, the grafted film was dried in a vacuum oven (10 mbar) at 80 °C for at least 2 h and weighed to determine the gravimetric graft level (GL).

Dry films were immersed in 10 v% of chlorosulfonic acid in DCM to convert styrene to styrene sulfonate groups. The reaction was carried out at room temperature for 4 hours. After completion of the reaction, the films were taken out of the solution and placed in water to quench any residual chlorosulfonic acid, followed by three washing steps with water. The films were then placed in a beaker containing ultra-pure water and left stirring overnight at 80 °C for hydrolysis.

Antioxidant doping

A sulfonated membrane sheet (~300 mg) was immersed into a 60 mL glass reactor that contained the Cu-porphyrin antioxidant (34.5 mg, 0.05 mmol, 1 eq.) and Et_3N (5.1 mg, 7 µL, 0.05 mmol, 1 eq.) in a mixture of DMF and H_2O (3:1, 50 mL in total). Then the reactor was placed under N_2 and stirred at room temperature for 15 min. Finally, the reactor was stirred at 65 °C for 18h. After the reaction was finished, the reaction mixture was cooled to room temperature. The membrane was successively washed with DCM and water and then placed for 1 h in a 1 M H_2SO_4 solution. After this protonation step the membrane was washed three times with water.

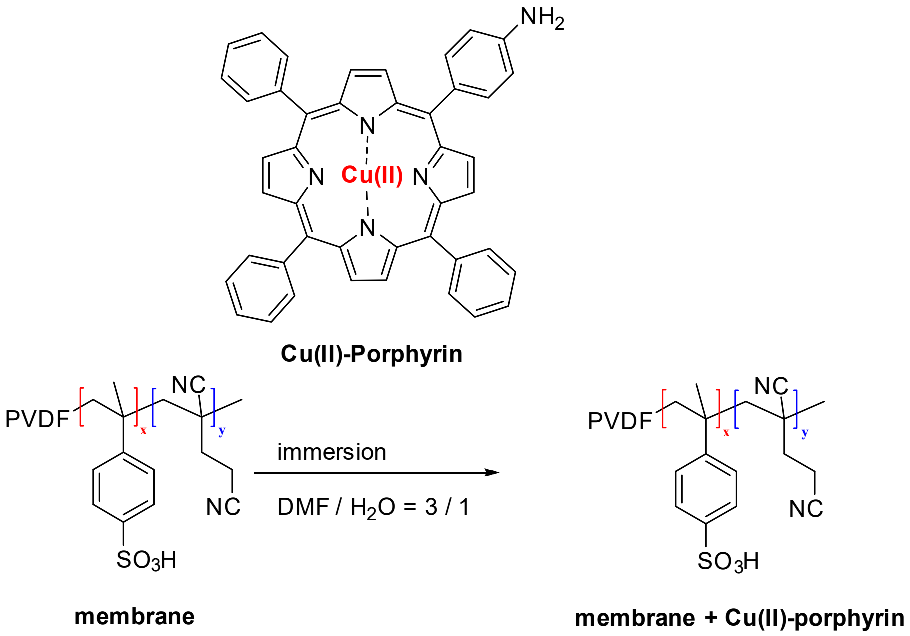

Scheme S1. Antioxidant doping reaction.

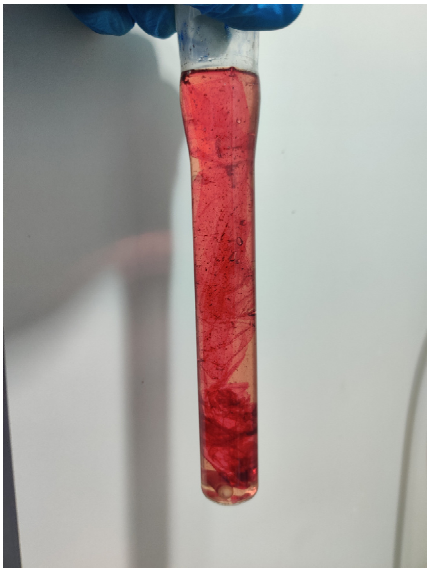

Figure S1. Color of the Cu(II)-porphyrin-containing PEM during the doping reaction.

Ion exchange capacity (IEC) determination

Ion exchange capacities were determined by acid-base titration. First, the membrane was exchanged overnight in 1 M KCl solution, to yield the potassium form and release protons into solution. The free protons in solution were determined by a single endpoint titration using a 877 Titrino plus (Metrohm AG, Switzerland) titrating unit with 0.05 M KOH as a titrant. Afterwards the membrane was taken out and rinsed three times with ultra-pure water to remove any excess salt. The membrane was dried under vacuum at 80 °C to obtain the dry mass which was corrected for the difference in molecular mass of the counter ion.

$$m_{\mathrm{dry,\,H^+}} = m_{\mathrm{dry,\,K^+}} - (\mathrm{M_{K^+}} - \mathrm{M_{H^+}}) * n(\mathrm{H^+}) \qquad [1]$$

m is the mass in g and *M* is the molar mass in g mol^{-1} for potassium or proton and *n* is the number of protons in mol. Three to five smaller pieces were cut from the same membrane and measured, or a single piece was measured in triplex to obtain the average IEC according to equation [2].

$$IEC = \frac{n(\mathrm{H^+})}{m_{\mathrm{dry,\,H^+}}} \qquad [2]$$

Proton conductivity measurements

Proton conductivities were measured at 25 °C in-plane with respect to the membrane. The measurement was conducted using a Bekktech BT-112 conductivity cell (Scribner, USA), which consists of a 4-point probe setup with Pt-wires. The cell was connected to a LCR meter (GWInstek LCR-6100) and the resistance, *R*, was measured at a frequency of 1 kHz and a perturbation amplitude of 100 mV. Throughout the measurement, the membranes were fully hydrated by immersion in ultra-pure water (18.2 MΩ). The wet thickness of the membrane was measured using a MT1281 Heidenhain thickness gauge and averaging over three different measurement spots. The conductivity was calculated using equation [3].

$$\sigma = \frac{l}{d \cdot R \cdot t} \qquad [3]$$

σ is the conductivity in S cm^{-1}, *l* is the distance of the voltage sense probes in mm, *d* is the sample width in cm, *R* is the resistance at 1 kHz in kΩ, and *t* is the membrane thickness in µm.

Membrane electrode assembly (MEA) preparation and fuel cell assembly

Prior to fuel cell testing selected membranes were fabricated into membrane electrode assemblies. The procedure was as follows. Wet membrane was taken out and dried briefly between two pieces of dust-free paper. The membrane was then placed in-between two 25 µm thick polyethylene napthalate (PEN) subgaskets and two commercial Pt-coated gas diffusion electrodes featuring a PFSA-based binder (Johnson Matthey ELE 0244, 0.4 mg Pt cm^{-2}) and directly assembled into the cell. Two 100 µm thick polytetrafluoroethylene (PTFE) gaskets were used to ensure proper compression.

The fuel cell used for the experiments is designed in-house and consists of two graphite plates with a parallel flow field, two Au-plated copper current collectors, and two aluminum compression plates that have 6 mm Swagelok connections. The active area is 16 cm^2 with the subgasket. For all experiments a co-flow configuration was used. The compression of the MEA was measured to be roughly 2.5 MPa using Fuji Film pressure sensitive film.

Fuel cell testing

The fuel cells were operated and tested on a fuel cell test bench that was designed and built in-house. LabVIEW software was used to monitor and control the different devices and parameters in the test bench. Gas flow rates were controlled using a mass flow controller (Brooks Instrument, the Netherlands), water flow was controlled using a liquid flow controller (Bronkhorst, the Netherlands). The gas and water flows were mixed using a controlled evaporator mixer (CEM, Bronkhorst, the Netherlands) to control the gas relative humidity. The high frequency resistance (HFR) was measured using a Hioki 3561-01 AC Milliohm Hi-Tester/Battery Tester. A load bank (Agilent system DC electronic load, N3300A) was used to draw current from the cell. For H_2 crossover measurements a DC power supply was used (Agilent, E3633A). The cell temperature was measured using a K-type thermocouple that was inserted into the middle of the cathode flow field.

For every newly assembled cell a hydrogen leak test was performed to make sure the cell was leak tight. After passing the leak test, the procedure consisted of: 1) conditioning protocol, 2) polarization curves, 3) hydrogen crossover measurement, 4) accelerated stress test.

Cell conditioning procedure

Every newly assembled cell was conditioned before any measurements. Fully humidified H_2 and O_2, with a flow rate of 200 sccm, were supplied to the anode and cathode, respectively. The cell was kept at 80 °C with a back pressure of 2.5 bar_a and a current density of 0.5 A cm^{-2} until a stable cell potential and HFR was reached. During the conditioning period, the cell voltage increased and the HFR decreased, until no further changes could be observed.

Polarization curve

Polarization curves were measured galvanostatically, directly after the break-in procedure. The current was increased from 0 to 0.25 A cm^{-2} in steps of 0.0625 A cm^{-2} and from 0.25 to 3.75 A cm^{-2} in steps of 0.25 A cm^{-2}. H_2 and O_2 were fed to the anode and cathode throughout, with a stoichiometry of 1.5 and a minimum flow rate of 200 sccm. The cell was maintained at 80 °C with a back pressure of 2.5 bar_a with 100% RH at the inlet.

Staircase voltammetry

The hydrogen gas crossover current was measured using staircase voltammetry. The fuel cell anode (counter and reference electrode) was placed under H_2, whereas the fuel cell cathode (working electrode, WE was placed under N_2 and left to equilibrate to reduce all Pt oxides to Pt on the WE. Typically, an OCV of around 0.1 V for an intact membrane was obtained. The cell potential was then set to 0.8 V and decreased stepwise to 0.2 V in steps of 0.1 V, and afterwards increased stepwise back to 0.8 V in steps of 0.2 V. The duration of each step was 60 seconds to ensure that a stable current was obtained. The average current of the last 15 seconds was recorded and plotted as a function of cell potential. The limiting crossover current was determined by extrapolating the current to the OCV.

Accelerated stress tests

Hydrocarbon-specific AST: Accelerated stress tests were performed at OCV (load bank cables disconnected to avoid leakage current) with fully humidified (100% RH) H_2 and O_2 and a back pressure of 2.5 bar_a to ensure a high oxygen partial pressure. The cell temperature was kept at 80 °C.

DOE-like AST: OCV hold test performed at 90 °C cell temperature, at ambient pressure, 30% RH at the inlet for H_2 and air.

Membrane post-test analysis

After the fuel cell testing protocol, the cell was purged with dry N_2 and cooled down to room temperature. The cell was disassembled and the MEA was removed. To remove the GDEs the MEA was immersed in a mixture of 50 v% water and ethanol which caused the MEA to delaminate. All samples were dried in a vacuum oven (80 °C, < 10 mbar) for at least 2 hours before the follow-up experiments.

Fourier Transform Infrared Spectroscopy (FT-IR)

FT-IR spectra were recorded in-house using a Bruker vortex 70v apparatus. Measurements were done under vacuum to suppress water absorption of the membranes. Every measurement consisted of 18 scans that were averaged. Scanning was done from 400 to 4000 cm^{-1} with a resolution of 2 cm^{-1}. An automatic background (vacuum) subtraction was done prior to analyzing the data.

X-ray fluorescence spectroscopy (XRF)

XRF was performed with an EDAX Orbis PC XRF. Membrane samples were vacuum dried at 80 °C before analysis. Analysis was performed at ambient conditions using an accelerating voltage of 35 kV and a current of 500 μA. Copper and sulfur were quantified from the area of the respective emission bands which are 8.1 and 8.9 eV for Cu K_{α} and K_{β} emission, respectively, and 2.31 eV for S K_{α} emission. The S intensity was used to normalize the data. The equipment was calibrated with high purity known elements.

Determination of the relative Cu(II) content by XRF

XRF cannot determine the speciation of Cu, chemical intuition suggests that it is complexed as Cu(II) by the porphyrin host, otherwise wash-out would occur during the fuel cell tests. Therefore, we refer to it as 'Cu-content'. Since no certified polymer reference materials exist, strong matrix effects were expected for the quantification of the Cu-content. Therefore, the concentrations can only be compared relatively and not as absolutes. Data are given as ppm but should be considered as arbitrary units (AU) (**Table 3**). The ratios of the abundance of elements S and Cu were obtained (**Table 3**). The IEC is directly related to the S-content of the membranes. By measuring the IEC of the respective membrane samples BOT and EOT (**Tables 1 and 2**), and multiplying it with the ratios of the abundance of elements S and Cu, we could estimate the Cu concentration. The shown error is the spot-to-spot deviation of three to five different locations on the membrane.

REFERENCES

(1) De Wild, T.; Wurm, J.; Becker, P.; Günther, D.; Nauser, T.; Schmidt, T. J.; Gubler, L.; Nemeth, T. A Nature-Inspired Antioxidant Strategy Based on Porphyrin for Aromatic Hydrocarbon Containing Fuel Cell Membranes. *ChemSusChem* **2023**, *16* (21), e202300775. https://doi.org/10.1002/cssc.202300775.
